# Low CCL-21 expression associates with unfavorable postoperative prognosis of patients with metastatic renal cell carcinoma

**DOI:** 10.18632/oncotarget.12827

**Published:** 2016-10-23

**Authors:** Ying Xiong, Li Liu, Jiajun Wang, Wei Xi, Yu Xia, Qi Bai, Yang Qu, Qilai Long, Jiejie Xu, Jianming Guo

**Affiliations:** ^1^ Department of Urology, Zhongshan Hospital, Fudan University, Shanghai, China; ^2^ Department of Biochemistry and Molecular Biology, School of Basic Medical Sciences, Fudan University, Shanghai, China

**Keywords:** CCL-21, metastatic renal cell carcinoma, prognostic factor, overall survival, progression-free survival

## Abstract

**Background:**

Chemokine (C-C motif) ligand 21 (CCL21), a ligand of the chemokine (C-C motif) receptor 7, has recently been identified as an immuno-based anti-cancer molecule for its dendritic cells and T lymphocytes chemoattractant function. The aim of this study was to investigate prognostic values of CCL21 expression in metastatic renal cell carcinoma patients treated with targeted therapy.

**Methods:**

This study included 111 patients with metastatic renal cell carcinoma receiving targeted therapy. CCL21 expression was analyzed by immunohistochemistry on tissue microarrays. Prognostic value of tumoral CCL21 expression and patients clinical outcomes were evaluated.

**RESULTS:**

Kaplan-Meier method showed that low CCL21 expression was associated with shorter patient overall survival and progression-free survival (overall survival, *P* = 0.005; progression-free survival, *P* = 0.044). Further stratified analysis showed that low CCL21 expression was significantly associated with shorter overall survival in clear cell renal cell carcinoma patients (*P =* 0.017) and patients treated with sorafenib (*P* = 0.009). Low CCL21 expression was also an adverse independent risk factor for overall survival (hazard ratio, 2.106; 95% CI, 1.286-3.450; *P =* 0.003) and progression-free survival (hazard ratio 1.617; 95%CI 1.060-2.465; *P* = 0.026) in multivariate analyses. CCL21 expression was significantly associated with treatment best response to targeted therapy (*P* = 0.009). This molecule could also be combined with Heng risk model to increase its overall survival predictive accuracy.

**Conclusion:**

Low CCL21 expression was a potential independent adverse prognostic biomarker for overall survival and progression-free survival for metastatic renal cell carcinoma patients treated with targeted therapy.

## INTRODUCTION

Renal cell carcinoma (RCC) is the eighth most common cancer in the world and accounts for 2% to 3% of all adult malignancies [1]. Around 20% to 40% patients would develop progressions or metastasis even after undergoing curative nephrectomy and 25% of patients with RCC have metastasis at first diagnosis [2, 3]. In recent years, the management of metastatic renal cell carcinoma (mRCC) has been revolutionized, with targeted therapies such as tyrosine kinase inhibitors (TKIs) superseding cytokine therapy (interferon-α and interleukin-2) [4]. Compared with Memorial Sloan Kettering Cancer Center(MSKCC) score, the Heng risk score does not show great improvement in predictive accuracy, indicating that prognosis prediction for mRCC patients with clinical factors alone was not enough [5]. Searching for biomarkers to predict the prognosis of mRCC patients treated with TKIs are of critical importance.

Chemokines belong to a superfamily of small chemotactic cytokines which induce the migration and activation of leukocytes [6]. Chemokine (C-C motif) ligand 21 (CCL21) is a lymphoid chemokine mainly produced by lymphatic vessels, stromal cells in the spleen and appendix, high endothelial venules in lymph nodes, Peyer's patches and some cancer cells [7, 8]. CCL21 is a chemoattractant for dendritic cells and T lymphocytes through their chemokine (C-C motif) receptor 7 (CCR7) expressions [9]. One research in renal cell carcinoma found that local expression of CCL21 by tumor cells led to accumulation of mature dendritic cells and proliferating T-cells at the margin, exhibiting a local anti-tumor immune response [10]. The impact of CCL21 expression on the prognosis of mRCC patients and treatment response to targeted therapy still remains unclear.

In this study, we investigated the expression of tumoral CCL21 in a large cohort of mRCC patients treated with sunitinib or sorafenib through immunohistochemistry on tissue microarrays. We analyzed the impact of CCL21 expression on patients’ overall survival (OS), progression-free survival (PFS) and treatment response to targeted therapy.

## RESULTS

### CCL21 staining and its association with pathological characteristics

CCL21 expression was analyzed by immunohistochemical staining on tissue microarrays. CCL21 expressions were mostly found in cytoplasm of tumor cells (Figure S1A and S1B). Inter-observer agreement of CCL21 IOD scores from the two observers was acceptable according to the kappa value 0.762, and then they were again averaged as the final IOD. We illustrated the smooth estimated HR of CCL21 expression (+1 IOD score) on patient OS (Figure S1C). IOD = 17138 was chosen as cutoff points according to minimum p value method with X-tile for clinical use and further analyses [11]. There were 60 patients (54.1%) grouped as CCL21 low expression and 51(45.9%) as CCL21 high expression.

**Figure 1:**
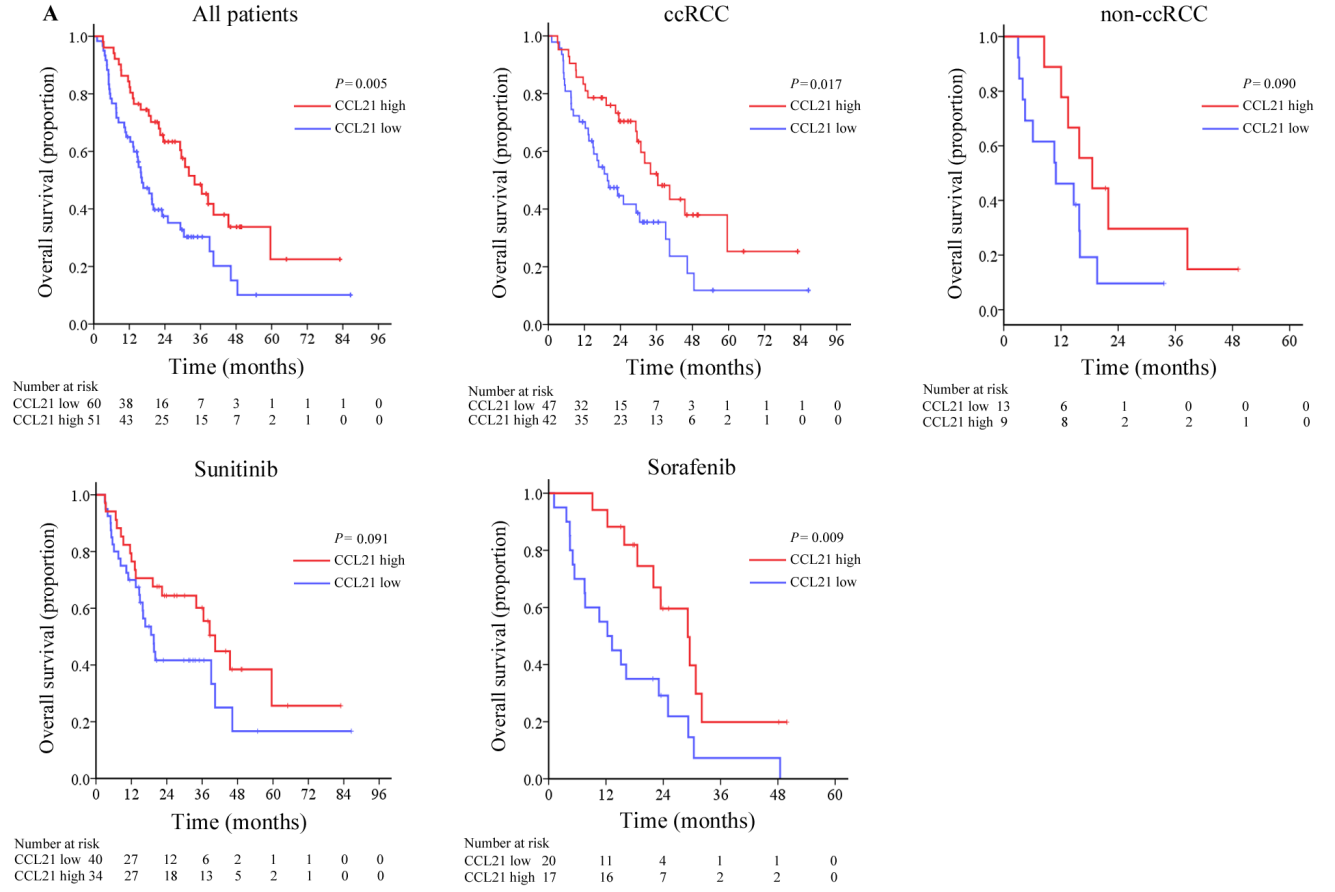
Overall survival (OS) analyses of patients with metastatic renal cell carcinoma (mRCC) based on CCL21 expression Kaplan-Meier analyses of OS in all patients (*n* = 111) **A**.; and in ccRCC patient group (*n* = 89) **B**.; in non-ccRCC patient group (*n* = 22) **C**.; in the group of patients receiving sunitinib (*n* = 74) **D**.; in the group of patients treated with sorafenib (*n* = 37) **E**.

Basic clinicopathological characteristics of the 111 patients were shown in Table 1. All patients had received partial, radical or cytoreductive nephrectomy. The average age at the time when patients first received targeted therapy was 57.49 years old (range 14 to 78). The histological subtype of 89 patients (80.2%) was clear cell renal cell carcinoma. There were 74 patients (66.7%) treated with sunitinib and 37 patients (33.3%) treated with sorafenib. There were 23 (20.7%) patients in the favorable risk group, 60 (54.1%) in the intermediate risk group and 28 (25.2%) in the poor risk group according to Heng risk criteria. The median follow-up time was 19.60 months (range 1.13 to 86.57). CCL21 expression was significantly associated with number of metastatic sites (*P =* 0.020) and Heng's risk model (*P =* 0.020).

**Table 1:** Clinical characteristics of patients according to CCL21 expression

Characteristics	Patients		CCL21 expression		
	n	%	low	high	*P*-value
All patients	111	100	60	51	
Gender					0.901†
Female	32	28.8	17	15	
Male	79	71.2	43	36	
Prior nephrectomy					
Yes	111	100			
No	0	0			
KPS					0.061†
≥80	82	73.9	40	42	
<80	29	26.1	20	9	
Histology					0.096‡
Clear-cell	89	80.2	47	42	
Non-clear cell	22	19.8	13	9	
papillary	15	13.5	7	8	
bellini	2	1.8	2	0	
chromophobe	2	1.8	1	1	
unclassified	3	2.7	3	0	
Fuhrman grade					0.609‡
1	2	1.8	1	1	
2	54	48.6	30	24	
3	41	36.9	21	20	
4	7	6.3	2	5	
Heng's risk model					0.020‡
Low risk	23	20.7	10	13	
Mediate risk	60	54.1	29	31	
High risk	28	25.2	21	7	
Number of disease sites					0.020
1	77		36	41	
≥2	34		24	10	
Sites of disease					
lung	83	74.8			
bone	18	16.2			
brain	2	1.8			
other sites	13	11.7			
Treatment					1.000†
sunitinib	74	66.7	40	34	
sorafenib	37	33.3	20	17	

† χ^2^ test or Fisher's exact test, ‡Cochran-Mantel-Haenszel χ^2^ test, *P*-value<0.05 was regarded as statistically significant. Abbreviations: KPS, Karnofsky performance status

### Low CCL21 expression indicates poor OS in mRCC patients

Kaplan-Meier survival analysis was performed to compare OS according to CCL21 expression. Patients with low CCL21 expression had significantly worse OS than those with high CCL21 expression as is shown in Figure 1A (*P =* 0.005). Univariate analysis showed that both IOD score of CCL21 expression as a continuous and dichotomous variable were significantly associated with OS (continuous, *P =* 0.008; dichotomous, *P =* 0.006) (Table 2). Multivariate analysis was further performed using CCL21 expression as a dichotomous variable and low CCL21 expression was still an adverse independent prognosticator for OS (HR, 2.106, 95% CI, 1.286-3.450, *P =* 0.003). Variables with p value lower than 0.1 in univariate analysis were included in the multivariate analysis.

**Table 2:** Univariate and multivariate analysis of characteristics associated with overall survival

Variables	Univariate analysis			multivariate analysis		
	Hazard Ratio	95%CI	*P*-value^†^	Hazard Ratio	95%CI	*P*-value^†^
Age, years	0.986	0.967-1.004	0.123			
Gender						
Male *vs* Female	0.927	0.616-1.702	0.927			
Histology						
non-ccRCC *vs* ccRCC	2.143	1.244-3.693	**0.006**	2.906	1.633-5.172	**<0.001**
Fuhrman grade			0.377			
2 *vs* 1	2.772	0.370-20.769	0.321			
3 *vs* 1	3.815	0.512-28.426	0.191			
4 *vs* 1	4.059	0.467-35.237	0.204			
Treatment						
sorafenib *vs* sunitinib	1.616	0.996-2.620	**0.052**	1.702	1.023-2.834	**0.041**
KPS						
<80 *vs* ≥80	1.401	0.827-2.374	0.210			
Time from diagnosis to targeted therapy						
<1 *vs* ≥1	1.465	0.918-2.339	0.110			**-**
Hemoglobin						
<LLN *vs* ≥ LLN	2.151	1.332-3.474	**0.002**	1.993	1.200-3.312	**0.008**
Serum corrected calcium						
>ULN *vs* ≤ULN	1.914	0.944-3.882	0.072			
Neutrophils						
>ULN *vs*≤ULN	1.550	0.893-2.689	0.119			
Platelets						
>ULN *vs* ≤ULN	2.070	1.207-3.551	**0.008**	1.943	1.120-3.369	**0.018**
CCL21 expression						
Low *vs* High	1.965	1.215-3.179	**0.006**	2.106	1.286-3.450	**0.003**
IOD scores of CCL21 staining	1.000	1.000-1.000	0.008			

KPS=Karnofsky performance status; LLN=lower limit of normal; ULN= upper limit of normal; CI=confidence interval; OS= overall survival; RFS= recurrence-free survival; ^†^Data obtained from the Cox proportional hazards model, *P*-value <0.05 was regarded as statistically significant

We also identified histology of non-ccRCC (*P <* 0.001), treatment of sorafenib (*P =* 0.041), hemoglobin less than the lower limit of normal (*P =* 0.008), platelets greater than the ULN (*P =* 0.018) as adverse prognostic factors for OS (Table 2). Kaplan-Meier survival analysis was performed in subgroups depending on histological type and systematic treatment. Low CCL21 expression still indicated shorter OS in patients with clear cell renal cell carcinoma (*P =* 0.017) and patients treated with sorafenib (*P =* 0.009) (Figure 1B, 1C, 1D, 1E). Univariate subgroup analyses showed that CCL21 expression was a risk factor in ccRCC patients (*P* = 0.019), patients treated with sorafenib (*P* = 0.012), patients with one metastatic site (*P* = 0.044) and patients with no lymph node involvement (*P* = 0.019) (Table S2).

### Extension of Heng's risk model with CCL21 expression

**Figure 2:**
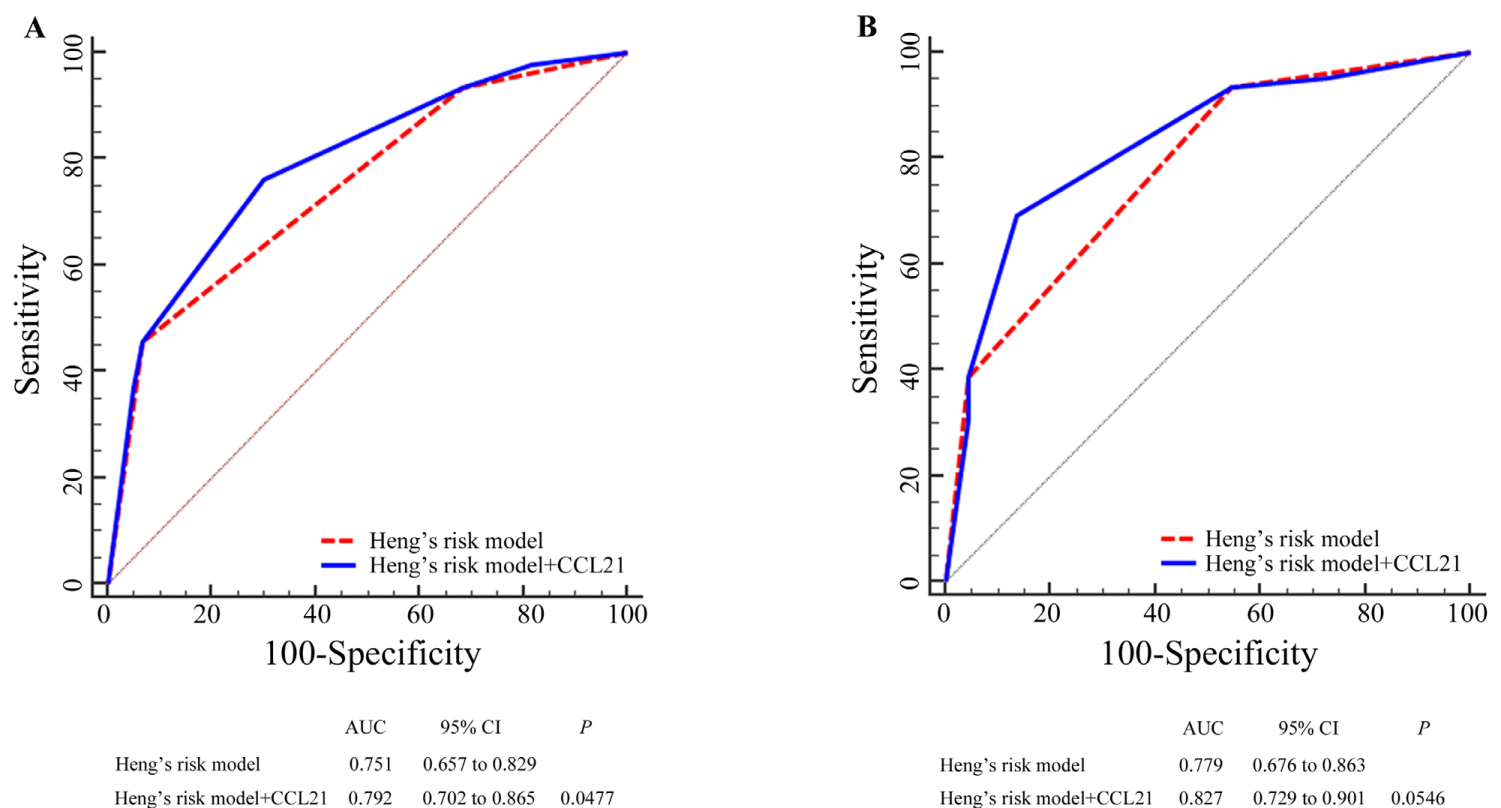
Receiver operating characteristic curve (ROC) analyses for the prediction of overall survival (OS) in mRCC patients Sensitivity and specificity to predict OS at the time of 18-month follow up **A**. and 36-month follow up **B**. using Heng's risk model and CCL21 expression combined and only Heng's risk model. *P* values show the area under the ROC curves (AUC) of the combined CCL21expression and Heng's risk model *versus* AUCs of Heng's risk model alone

We performed ROC analyses at the time of 18-month and 36-month follow ups by integrating CCL21 expression with Heng's risk model (Figure 2). The combination of CCL21 expression (high/low) to the Heng's risk model (favorable/intermediate/poor) showed better prognostic power. At the 18-month follow up, the combined model (AUC = 0.792, 95%CI: 0.702-0.865) performed better than Heng's risk model alone (AUC = 0.751, 95%CI: 0.657-0.829) and reached statistical significance (*P =* 0.0477). At the 36-month follow up, the combined model performed better as well (CCL21+Heng's risk model: AUC = 0.827, 95%CI: 0.729-0.901; Heng's risk model: AUC = 0.779, 95%CI: 0.676-0.863) and reached borderline statistical significance (*P =* 0.0546). However, combining CCL21 expression with Heng's risk model did not increase predictive accuracy for PFS.

### Impact of CCL21 expression on treatment response and PFS in mRCC patients

**Figure 3:**
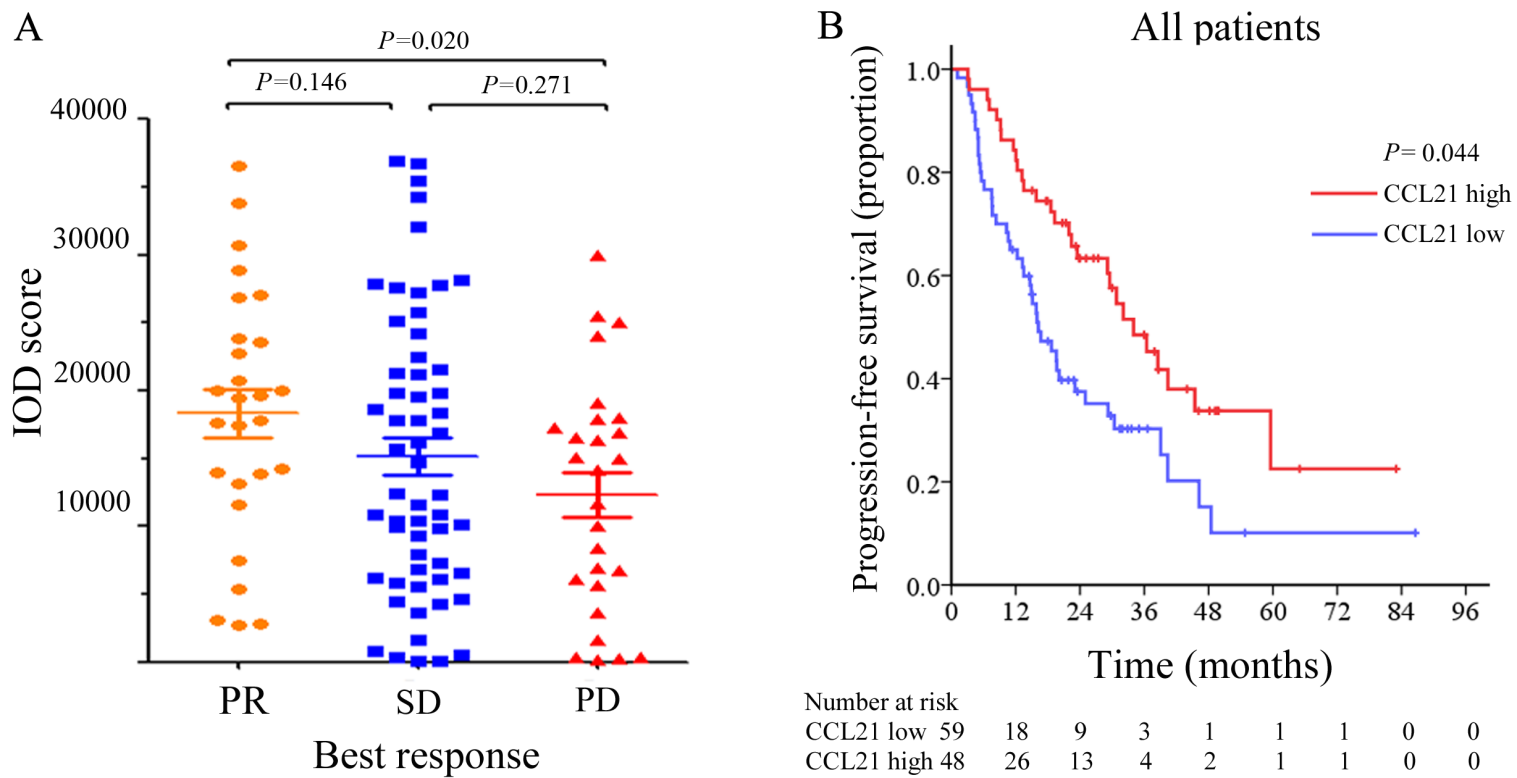
CCL21 staining intensity distribution and progression-free survival (PFS) analyses of mRCC patients IOD scores of CCL21according to mRCC patients’ best drug response **A**.. Kaplan-Meier analyses of PFS in all patients **B**..

During the follow up time, 91 of the 107 patients (85.1%) developed disease progression and four patients were excluded due to incomplete information. Treatment responses were evaluated based on the RECIST 1.1 criteria. In this cohort, twenty-seven patients reached partial response (PR), fifty-seven patients had stable disease (SD), twenty-three patients had progressive disease (PD). The objective response rate for patients with low and high CCL21 expression were 16.9% and 35.4% respectively. As is shown in Figure 3A, IOD scores of CCL21 expression were significantly higher in PR patients group compared with PD group (*P =* 0.020). We found that low CCL21 expression is significantly associated with adverse treatment best responses (*P* = 0.009) (Table S1).

The median PFS for the two groups were 6.3 months and 17.7 months. Kaplan Meier analysis showed that patients with low CCL21 expression had shorter PFS (*P =* 0.044) (Figure 3B). After being stratified according to targeted therapy, CCL21 expression was still significantly associated with treatment best responses in mRCC patients receiving sorafenib (*P* = 0.010). In sunitinib subgroup the correlation did not reach statistical significance (*P* = 0.177). Multivariate analysis was further performed, and results showed that CCL21 expression was an independent risk factor for PFS (hazard ratio 1.617; 95%CI 1.060-2.465; *P* = 0.026) (Table 3). Univariate subgroup analyses showed that CCL21 was still a risk factor in patients treated with sorafenib (*P* = 0.031) (Table S2).

**Table 3:** Univariate and multivariate analysis of characteristics associated with progression-free survival

Variables	Univariate analysis			multivariate analysis		
	Hazard Ratio	95%CI	*P*-value^†^	Hazard Ratio	95%CI	*P*-value^†^
Age, years	0.989	0.972-1.006	0.195			
Gender						
Male *vs* Female	1.311	0.813-2.115	0.266			
Histology						
non-ccRCC *vs* ccRCC	1.601	0.979-2.619	**0.061**	1.706	1.040-2.799	**0.034**
Fuhrman grade			0.834			
2 *vs* 1	1.509	0.360-6.327	0.574			
3 *vs* 1	1.587	0.378-6.661	0.528			
4 *vs* 1	1.994	0.408-9.737	0.394			
Treatment						
sorafenib *vs* sunitinib	1.616	0.882-2.097	0.164			
KPS						
<80 *vs* ≥80	1.428	0.900-2.267	0.130			
Time from diagnosis to targeted therapy						
<1 *vs* ≥1	1.215	0.805-1.836	0.354			-
Hemoglobin						
<LLN *vs* ≥ LLN	1.458	0.962-2.210	**0.076**			
Serum corrected calcium						
>ULN *vs* ≤ULN	2.598	1.354-4.981	**0.004**	2.991	1.544-5.796	**0.001**
Neutrophils						
>ULN *vs*≤ULN	1.015	0.612-1.684	0.954			
Platelets						
>ULN *vs* ≤ULN	1.546	0.948-2.522	**0.081**			
CCL21 expression						
Low *vs* High	1.533	1.008-2.330	**0.046**	1.617	1.060-2.465	**0.026**

KPS=Karnofsky performance status; LLN=lower limit of normal; ULN= upper limit of normal; CI=confidence interval; OS= overall survival; RFS= recurrence-free survival; †Data obtained from the Cox proportional hazards model, *P*-value <0.05 was regarded as statistically significant

## DISCUSSION

In this study, we detected the tumoral CCL21 expression using 111 specimen tissues of mRCC patients treated with targeted therapies. Tumors with low CCL21 expression were more likely to metastases to more than one organ. Kaplan Meier analysis showed that the impact of CCL21 expression on OS was more profound in ccRCC patients and patients treated with sorafenib. Low CCL21 expression was an adverse independent risk factor for OS and PFS in mRCC patients treated with targeted therapies in multivariate analysis. ROC analyses showed that CCL21 expression level could be combined with Heng's risk model to improve its OS predictive efficacy. Besides, our study suggested that patients with low tumoral CCL21 expression might benefit less from TKIs treatment, since it was associated with shorter PFS and worse treatment response, which was more prominent in the sorafenib group. Thus, we believe CCL21 is a potential biomarker for predicting prognosis in mRCC patients treated with targeted therapies.

Numerous evidences have shown that CCL21 could boost anti-tumor immunity. CCL21 is able to attract and colocalize naive lymphocytes and DC, thus promoting cognate T cell activation. CCL21 has the potential to induce effective anti-tumor immunity and suppress immune tolerance [12]. Injection of CCL21 can also generate systemic immune responses, increase infiltration of DC and T lymphocyte effectors and reduced myeloid derived suppressor cells as well as T regulatory cells [13, 14]. At University of California Los Angeles, a phase 1 clinical trial was conducted in non-small cell lung cancer patients to evaluate the safety and clinical efficacy of the intratumoral administration of DC-CCL21 [15].

Most studies identify CCL21 as a tumor-suppressing molecule, but there are also some studies show that CCL21/CCR7axis promotes growth and metastasis of many tumor types including melanomas, breast, thyroid, colon, head, and neck cancers [16–21]. The prognostic value of CCL21 also varies with different type of cancers [22–24]. Moreover, some limitations remained in this study. This was a retrospective study and the sample size was relatively small especially in subgroup analyses. Intratumoral heterogeneity cannot be avoided as well though we have taken two cores from each tumor block. Besides, the patients were enrolled from one single institution and composed of one ethnicity; further external validations of the CCL21 expression cut-off point choosing and its prognostic and predictive value should be performed. Patients took sunitinib and sorafenib as first-line therapy in our study because other targeted agents were not available at the time in China.

In conclusion, we have identified that low CCL21 expression was significantly associated with poor prognosis and drug response in patients treated with targeted therapy. The prognostic accuracy of Heng's risk model was increased if it was combined with CCL21 expression.

## METERIALS AND METHODS

### Patient selection

A total of 138 mRCC patients who were treated with sunitinib or sorafenib at the Department of Urology, Zhongshan Hospital, Fudan University between Mar 2005 and Jun 2014 were screened for this study. Patients were retrospectively included if they met the criteria of having pathologically proved mRCC, being treated with sunitinib or sorafenib as first-line targeted therapy, possessing available Formalin Fixed Paraffin Embedded (FFPE) specimen of tumor mass (≥1cm^3^) and detailed follow up information. Patients were excluded if they had other malignant tumors before or histories of former targeted therapy. Samples with over 80% necrotic or hemorrhagic area and patients with missing follow-up, imaging or laboratory data were also excluded. In the end, 111 patients were selected for this study. The Clinical Research Ethics Committee of Zhongshan Hospital, Fudan University (Shanghai, China) approved this study with the approval number B2015-030 and informed consent was obtained from each patient.

### Data collectioin

The primary outcome was OS, which was calculated from the time of therapy initiation to the time of death or last follow up. PFS was defined as the time from therapy initiation to the time of progression, following the RECIST 1.1 criteria [25], or to the last follow up time December 2015. The analysis of PFS excluded four patients with missing progression state.

All clinical, laboratorial, imaging and follow up data were collected retrospectively from medical records and electronic databases using uniform database templates. Two pathologists (Yuan J. and Jun H.) reviewed the H&E slides to reconfirm the histological subtype and Fuhrman grade. One urologist reassessed all the MRI and CT scans. Histological subtypes of RCC were identified according to 2014 EAU guidelines [26]. Fuhrman grade were recorded based on the 2012 ISUP consensus [27, 28]. Patients’ risks were stratified according to Heng's risk model [29].

### Immunohistochemistry

Immunohistochemical staining was performed on tissue microarray (two cores for one tumor block using primary antibodies against human CCL21 (Anti-CCL21 antibody, ab9851, abcam, diluted 1/500)) and visualization reagent (DakoEnVision Detection System) as previously described [30]. Antibody specificity was confirmed by immunochemistry and western blot. Olympus CDD camera, Nikon eclipse Ti-s microscope (×400magnification) and NIS-Elements F3.2 software were used to record the staining results. We took three independent shots with the strongest staining for each tumor core. The intensity of immunohistochemical staining of CCL21 was scored by two urologists unaware of the patients’ clinical features and outcomes using Image-Pro Plus version6.0 software (Media Cybernetics Inc., Bethesda, MD, USA). The kappa value was analyzed for evaluating inter-observer agreement. The pooled IOD mean of each patient's 2 cores (6 scans) was regarded as the staining intensity for each block. The IOD score from two observers were averaged again for final statistical analyses.

### Statistical analyses

Statistical analyses were performed using SPSS Statistics 21.0 (SPSS Inc., Chicago, IL), R software version 3.1.2 (R Foundation for Statistical Computing, Vienna, Austria) and X-tile (version 3.6.1; Robert L Camp, Yale University, CT, USA). The smooth estimates of hazard ratio (HR) of CCL21 IOD score on patient survival were displayed using R software, “phenoTest” package [31]. The cutoff point of CCL21 expression was selected according to “optimal p value method” with X-tile [11]. We then used Pearson's chi-square test, Fisher's exact test or Cochran-Mantel-Haenszel χ2 test to assess the correlation between clinicopathological parameters of the patients and CCL21 expression. Mann-Whitney U test was used to compare the IOD scores of patients with different drug best response. Kaplan-Meier analysis and log-rank test were performed to compare the survival between different patient groups. Numbers at risk were calculated at the beginning of each time period. The Cox proportional hazards regression model was used to perform univariate and multivariate analyses. Time dependent receiver operating characteristic (ROC) analysis was performed to analyze the prognostic value of Heng's risk model combined with CCL21 expression. All statistical tests were 2-sided and *P* < 0.05 was considered statistically significant.

## SUPPLEMENTARY MATERIALS FIGURES AND TABLES


