# Supplementary material for: Low CCL-21 expression associates with unfavorable postoperative prognosis of patients with metastatic renal cell carcinoma
Source: Oncotarget. 2016 Oct 23;8(15):25650–9. doi: 10.18632/oncotarget.12827 (PMC5421958; doi:10.18632/oncotarget.12827)
Supplement: Supplementary file 1 [file oncotarget-08-25650-s001.pdf]

Low CCL-21 expression associates with unfavorable postoperative prognosis of patients with metastatic renal cell carcinoma

Supplementary Material

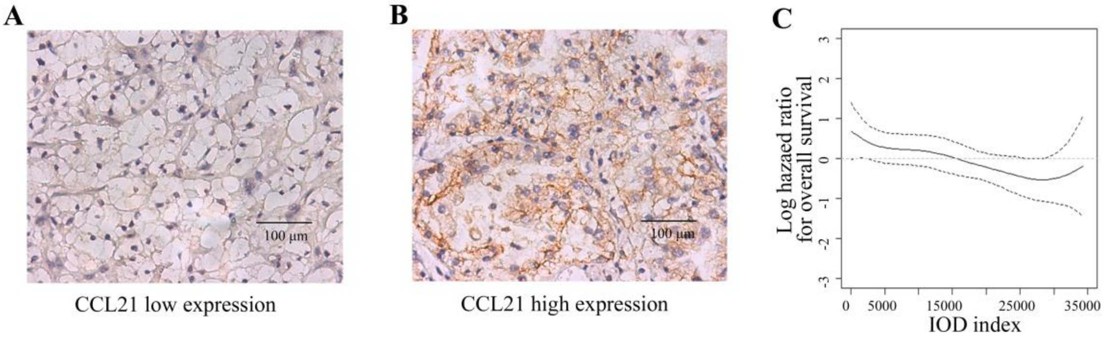

Figure S1. CCL21 expression in mRCC tissues and smooth estimates of hazard ratio (HR) (+1 IOD)

Representative immunohistochemical (IHC) images of mRCC tumor tissues with low CCL21 expression (A) and high CCL21 expression (B). Smooth estimates of HR (+1 IOD) showed a higher risk of death for patients with lower CCL21 expression (C).

Table S1.Best response* to targeted therapy according to CCL21 expression

	Tyrosine-kinase inhibitors		Sunitinib		Sorafenib	
	CCL21 low	CCL21 high	CCL21 low	CCL21 high	CCL21 low	CCL21 high
PR	10(16.9%)	17(35.4%)	9(23.1%)	11(34.4%)	1(5.0%)	6(37.5%)
SD	32(54.2%)	25(52.1%)	21(53.8%)	17(53.1%)	11(55.0%)	8(50.0%)
PD	17(28.8%)	6(12.5%)	9(23.1%)	4(12.5%)	8(40%)	2(12.5%)
P-value	0.009†		0.177†		0.010†	
RFS(months)	6.3	17.7	9.4	13.4	4.3	15.6

*evaluated according to RECIST version 1.1; †χ^2 test or Fisher's exact test; OS=overall survival, RFS=recurrence-free survival, PR=partial response, SD= stable disease, PD= progressive disease, RECIST=the Response Evaluation Criteria in Solid Tumors

Table S2: Hazard ratios for OS and PFS based on tumoral CCL21 expression in different subgroups (Low *vs* High)

Patient subgroups	OS (n=111)			PFS (n=107)		
	**Hazard Ratio**	**95%CI**	**P-value†**	**Hazard Ratio**	**95%CI**	**P-value†**
Histology						
Clear cell	1.941	1.114-3.383	**0.019**	1.521	0.943-2.453	0.085
Non-clear cell	2.334	0.847-6.431	0.101	1.586	0.660-3.813	0.302
Targeted therapy						
Sunitinib	1.692	0.914-3.132	0.094	1.247	0.744-2.089	0.402
Sorafenib	2.746	1.254-6.015	**0.012**	2.223	1.076-4.591	**0.031**
No. of metastatic sites*						
1	1.849	1.017-3.362	**0.044**	1.413	0.837-2.383	0.196
≥2	1.936	0.804-1.936	0.140	1.343	0.633-2.848	0.442
Lymph node involvement*						
No	1.999	1.119-3.569	**0.019**	1.545	0.937-2.549	0.088
Yes	2.091	0.857-5.100	0.105	1.848	0.816-4.185	0.141
Heng’s risk group						
Favorable	1.034	0.274-3.903	0.960	0.446	0.114-1.742	0.245
Intermediate	1.675	0.878-3.197	0.118	1.383	0.795-2.405	0.251
Poor	1.730	0.642-4.662	0.278	1.209	0.445-3.288	0.710

*At the time initializing targeted therapy; CI=confidence interval; OS= overall survival; PFS= progression free survival; †Data obtained from the Cox proportional hazards model, P-value <0.05 was regarded as statistically significant
